# Taste receptor type 1 member 3 enables western diet-induced anxiety in mice

**DOI:** 10.1186/s12915-023-01723-x

**Published:** 2023-11-06

**Authors:** Jae Won Song, Keon-Hee Lee, Hobin Seong, Dong-Mi Shin, Woo-Jeong Shon

**Affiliations:** 1 Department of Food and Nutrition, Seoul National University College of Human Ecology, Gwanak-Gu, Seoul, 08826 Republic of Korea; 2 Research Institute of Human Ecology, Seoul National University, Gwanak-Gu, Seoul, 08826 Republic of Korea

**Keywords:** Anxiety disorders, Western diet, Taste receptor type 1 member 3, Hypothalamus

## Abstract

**Background:**

Accumulating evidence supports that the Western diet (WD), a diet high in saturated fat and sugary drinks, contributes to the pathogenesis of anxiety disorders, which are the most prevalent mental disorders worldwide. However, the underlying mechanisms by which WD causes anxiety remain unclear. Abundant expression of taste receptor type 1 member 3 (TAS1R3) has been identified in the hypothalamus, a key brain area involved in sensing peripheral nutritional signals and regulating anxiety. Thus, we investigated the influence of excessive WD intake on anxiety and mechanisms by which WD intake affects anxiety development using wild-type (WT) and *Tas1r3* deficient (*Tas1r3*^−/−^) mice fed a normal diet (ND) or WD for 12 weeks.

**Results:**

WD increased anxiety in male WT mice, whereas male *Tas1r3*^−/−^ mice were protected from WD-induced anxiety, as assessed by open field (OF), elevated plus maze (EPM), light–dark box (LDB), and novelty-suppressed feeding (NSF) tests. Analyzing the hypothalamic transcriptome of WD-fed WT and *Tas1r3*^−/−^ mice, we found 1,432 genes significantly up- or down-regulated as a result of *Tas1r3* deficiency. Furthermore, bioinformatic analysis revealed that the CREB/BDNF signaling-mediated maintenance of neuronal regeneration, which can prevent anxiety development, was enhanced in WD-fed *Tas1r3*^−/−^ mice compared with WD-fed WT mice. Additionally, in vitro studies further confirmed that *Tas1r3* knockdown prevents the suppression of *Creb1* and of CREB-mediated BDNF expression caused by high levels of glucose, fructose, and palmitic acid in hypothalamic neuronal cells.

**Conclusions:**

Our results imply that TAS1R3 may play a key role in WD-induced alterations in hypothalamic functions, and that inhibition of TAS1R3 overactivation in the hypothalamus could offer therapeutic targets to alleviate the effects of WD on anxiety.

**Supplementary Information:**

The online version contains supplementary material available at 10.1186/s12915-023-01723-x.

## Background

Anxiety disorders are defined as persistent, uncontrollable, and disproportionate feelings of anxiety that interfere with normal functioning in daily life [1]. Anxiety disorders have the highest prevalence of any mental disorder worldwide and are the leading cause of global disease burden (ranked as the eighth leading cause of years lived with disability in 2019) [2, 3]. Anxiety disorders are multifactorial mental illnesses that are influenced by genetic and environmental factors, such as childhood experiences, socioeconomic status, smoking, and alcohol abuse [4–6]. Among external factors, diet, one of the key factors that affects people’s health in their daily lives, also affects anxiety [7]. Particularly, there is growing recognition that the Western diet (WD), which is rapidly increasing in consumption globally, can exacerbate anxiety [8, 9]. Several rodent and human studies suggest that neither diets high in sugar, nor fat-induced anxiety, always correlate with weight gain, nor does anxiety improve with weight loss [10, 11]. There are several limitations associated with lifestyle and weight control to relieve diet-induced mental disorders. There is also a lack of effective medications for anxiety, and the precise molecular mechanisms underlying WD-linked anxiety remain largely undetermined; further research is necessary.

The amygdala, hippocampus, medial prefrontal cortex, and hypothalamus form a network of brain structures involved in the neuropathophysiology of anxiety [12]. Among these brain regions, the hypothalamus is located close to the median eminence; this is composed of fenestrated capillaries so it can directly sense blood-borne molecules (e.g., nutrients and hormones) to regulate body homeostasis [13, 14]. This suggests that the hypothalamus is the primary site involved in the integration of peripheral nutritional signals and emotional responses. Studies of the pathogenesis of diet-induced anxiety as related to the principal features of the hypothalamus have focused on increased inflammation and hormonal dysregulation [15, 16]. However, hypothalamic neurogenesis modulated by diet has recently emerged as a new mechanism underlying diet-induced anxiety that affects the differentiation of anxiolytic neurons and hippocampal functions [17, 18].

Taste receptor type 1 member 3 (TAS1R3) is a G protein-coupled receptor (GPCR) involved in taste perception via the taste buds on the tongue [19]. TAS1R3 forms heterodimeric GPCR complexes such as TAS1R1/TAS1R3 (umami stimuli) and TAS1R2/TAS1R3 (sweet stimuli) to sense ligands [20]. TAS1R3 is reportedly expressed in various extraoral tissues, such as the epithelium of the gastrointestinal tract, pancreatic islet cells, and testes [21–23]. Furthermore, TAS1R3 signals are expressed more in neurons than in other brain cell types, with the highest expression in the hypothalamic paraventricular and arcuate nuclei compared to other brain regions, such as the cortex and hippocampus [24]. TAS1R3 is expressed in the hypothalamic regions, which are known to be involved in neuroendocrine control and hypothalamic neurogenesis, primarily mediating physiological and behavioral responses to nutrient fluxes in circulation [25, 26]. Although recent studies have shown that nutrient-sensing receptors expressed in the hypothalamus modulate diet-induced mood disorders, the role of TAS1R3 in WD-induced anxiety remains unknown [27, 28].

In this context, we hypothesized that nutrient-induced modulation of hypothalamic TAS1R3 is central to the regulation of WD-induced anxiety. To elucidate possible role of TAS1R3 in regulating behavior, we investigated *Tas1r3* deficient animals. We evaluated the influence of excessive WD intake on anxiety-related behavior and investigated the mechanisms through which WD intake affects anxiety development. Our findings uncover the previously unknown role of TAS1R3 in the context of WD-induced anxiety-like phenotype and may contribute to the development of preventive or therapeutic strategies for anxiety disorders.

## Results

### Male *Tas1r3*^*-/-*^ mice were protected against immobility and anxiety-like behavior induced by WD

**Fig. 1:**
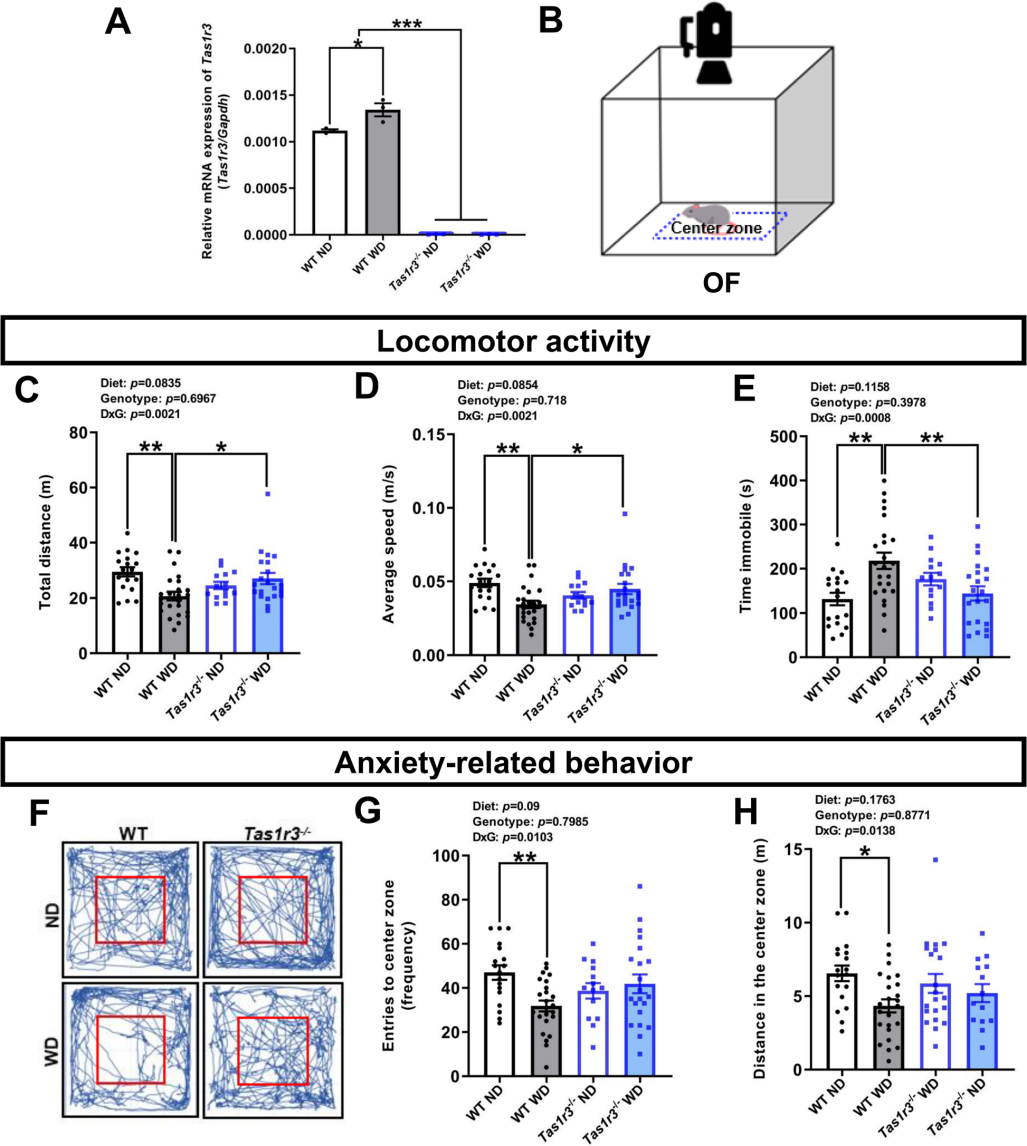
WD-induced immobility and anxiety-related behaviors in WT mice not exhibited in WD-fed *Tas1r3*^−/−^ mice*.* **A** *Tas1r3* mRNA expression in the hypothalamus of all groups. Two-way ANOVA followed by Tukey’s multiple comparison test: *p < 0.05, ***p < 0.001. *n* = 3 mice/group. **B** Schematic diagram of the OF test. The inner blue dotted line denotes the center zone. **C-E** Locomotor activities assessed by total distance traveled in the arena, average speed and time spent immobile. Two-way ANOVA followed by Tukey’s multiple comparison test: *p < 0.05, **p < 0.01. **F** Representative movement traces (blue lines) in OF chamber. **G**,**H** Anxiety-related behaviors measured by the frequency of entries to the center zone, and distance traveled in the center zone. Two-way ANOVA followed by Tukey’s multiple comparison test: *p < 0.05, **p < 0.01. WT ND: *n* = 18; WT WD: *n* = 24; *Tas1r3*^−/−^ ND: *n* = 14; *Tas1r3*^−/−^ WD: *n* = 21. All values are presented as the means ± SEM. ANOVA, analysis of variance; OF, open field; ND, normal diet; WD, western diet; WT, wild-type

To investigate the function of TAS1R3 in the behavioral performance of WD-fed mice, 8-week-old C57BL/6 WT and *Tas1r3*^-/-^ mice were fed an ND (10% fat diet with plain water) or WD (60% fat diet with 30% sucrose solution) for 12 weeks and subjected to a battery of behavioral tests. There was no significant difference between caloric intake between WT and *Tas1r3*^-/-^ mice (see Additional file 1: Fig. S1A-C). *Tas1r3* expression in the hypothalamus of WT mice was significantly increased by long-term WD intake (Fig. 1A). We first conducted an OF test (Fig. 1B) to assess the exploratory locomotor activity of mice exposed to the novel open field. WD-fed male WT mice showed relatively less travel in the arena and lower average velocity than ND-fed male WT mice and WD-fed male *Tas1r3*^-/-^ mice (Fig. 1C-D). Furthermore, the immobility time of male WT mice was significantly increased by WD, whereas WD-fed male *Tas1r3*^-/-^ mice had a similar range of locomotor activity to ND-fed male WT mice (Fig. 1E).

**Fig. 2:**
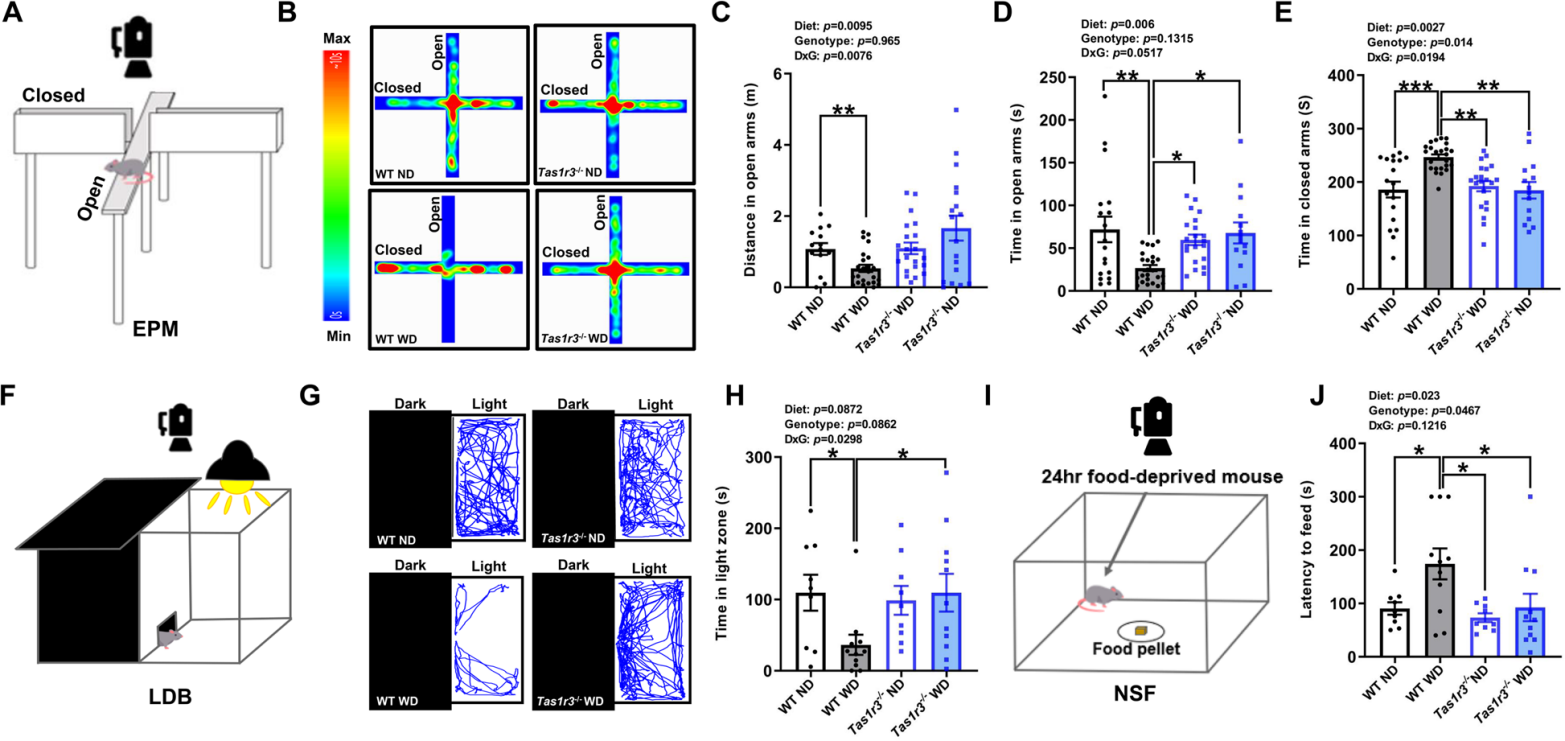
*Tas1r3* deficiency protects against the anxiety-like phenotypes induced by WD. **A** Schematic of EPM test. **B** Representative heatmaps of time spent in EPM. **C** Distance traveled in the open arms. Two-way ANOVA followed by Tukey’s multiple comparison test: **p < 0.01. **D** Time spent in open arms. Two-way ANOVA followed by Tukey’s multiple comparison test: *p < 0.05, **p < 0.01. **E** Time spent in closed arms. Two-way ANOVA followed by Tukey’s multiple comparison test: **p < 0.01, ***p < 0.001. WT ND: *n* = 18; WT WD: *n* = 24; *Tas1r3*^−/−^ ND: *n* = 14; *Tas1r3*^−/−^ WD: *n* = 21. **F** Schematic of LDB test. **G** Representative movement traces (blue lines) in LDB. **H** Time spent in light zone. Two-way ANOVA followed by Tukey’s multiple comparison test: *p < 0.05. WT ND: *n* = 9; WT WD: *n* = 11; *Tas1r3*^−/−^ ND: *n* = 9; *Tas1r3*^−/−^ WD: *n* = 11. **I** Schematic of NSF test. **J** Latency time to feed. Two-way ANOVA followed by Tukey’s multiple comparison test: *p < 0.05. WT ND: *n* = 9; WT WD: *n* = 11; *Tas1r3*^−/−^ ND: *n* = 9; *Tas1r3*^−/−^ WD: *n* = 11. All values are presented as the means ± SEM. ANOVA, analysis of variance; EPM, elevated plus maze; LDB, Light–dark box; ND, normal diet; NSF, novelty-suppressed feeding; WD, western diet; WT, wild-type

Regarding anxiety-related behavioral dimensions, WD-fed male WT mice were less inclined to encounter and travel the center zone than were ND-fed male WT mice, which is indicative of increased anxiety-like behavior. However, WD-fed male *Tas1r3*^-/-^ mice didn’t exhibit WD-induced anxiety-behaviors (Fig. 1F-H). The EPM test was used to measure additional anxiety-like behaviors (Fig. 2A). We found that WD-fed male WT mice displayed increased anxiety-like behavior, as evident by fewer distance traveled in the open arms and less time spent in the open arms compared with ND-fed male WT mice. Contrastingly, WD-fed male *Tas1r3*^-/-^ mice showed more time spent in the open arms compared with WD-fed male WT mice (Fig. 2D). In addition, WD-fed male WT mice were more likely to spend time in the closed arms than both ND-fed male WT and WD-fed male *Tas1r3*^-/-^ mice (Fig. 2E). In the LDB test, WD-fed male WT mice spent significantly less time in the light zone compared to ND-fed male WT mice, whereas WD-fed male *Tas1r3*^-/-^ mice spent more time in the light zone than WD-fed male WT mice (Fig. 2F-H). In line with the results of the OF, EPM, and LDB tests, WD-fed male WT mice exhibited longer latency time for eating pellets compared with ND-fed male WT mice and WD-fed male *Tas1r3*^-/-^ mice in NSF test (Fig. 2I-J).

After 12 weeks of high-calorie western diet intake, significant differences in body weight were observed between groups in male mice (see Additional file 1: Fig. S2A). Several studies showed that high-calorie western diet-induced obesity is a potential risk factor for anxiety [29, 30]. Therefore, we investigated whether WD-induced anxiety is dependent on weight gain. Linear regression analysis based on groups confirmed no significant association between body weights and anxiety-related parameters (see Additional file 1: Fig. S2B-H). To assess the effect of weight gain, we further analyzed the anxiety-related parameters using ANCOVA with body weights as the potential covariates. However, the ANCOVA model demonstrated that body weight was not a covariate significantly influencing anxiety-related behaviors (entries to center zone: *F* = 0.065, *p* = 0.8; distance in the center zone: *F* = 0.003, *p* = 0.958; distance in open arms: *F* = 2.414, *p* = 0.125; time in open arms: *F* = 1.501, *p* = 0.225; time in closed arms: *F* = 2.71, *p* = 0.104; time in light zone: *F* = 1.613, *p* = 0.212; latency to feed: *F* = 1.083, *p* = 0.305). These results suggest that the anxiety levels of male mice were independent of their body weights.

There were significant differences in body weight between groups in female mice, similar to the phenotype we observed in male mice (see Additional file 1: Fig. S3A). However, there was no significant difference in locomotor activities and anxiety-related behaviors between groups in female mice (see Additional file 2: Fig. S3B-E), likely due to reported sex-dependent effects in anxiety-related behaviors [31].

These results suggest that consumption of WD can lead to increased anxiety in WT mice. However, *Tas1r3*^*-/-*^ mice were protected from the WD-induced anxiety.

### *Tas1r3* deficiency largely alters gene expression in the hypothalamus of WD-fed mice

**Fig. 3:**
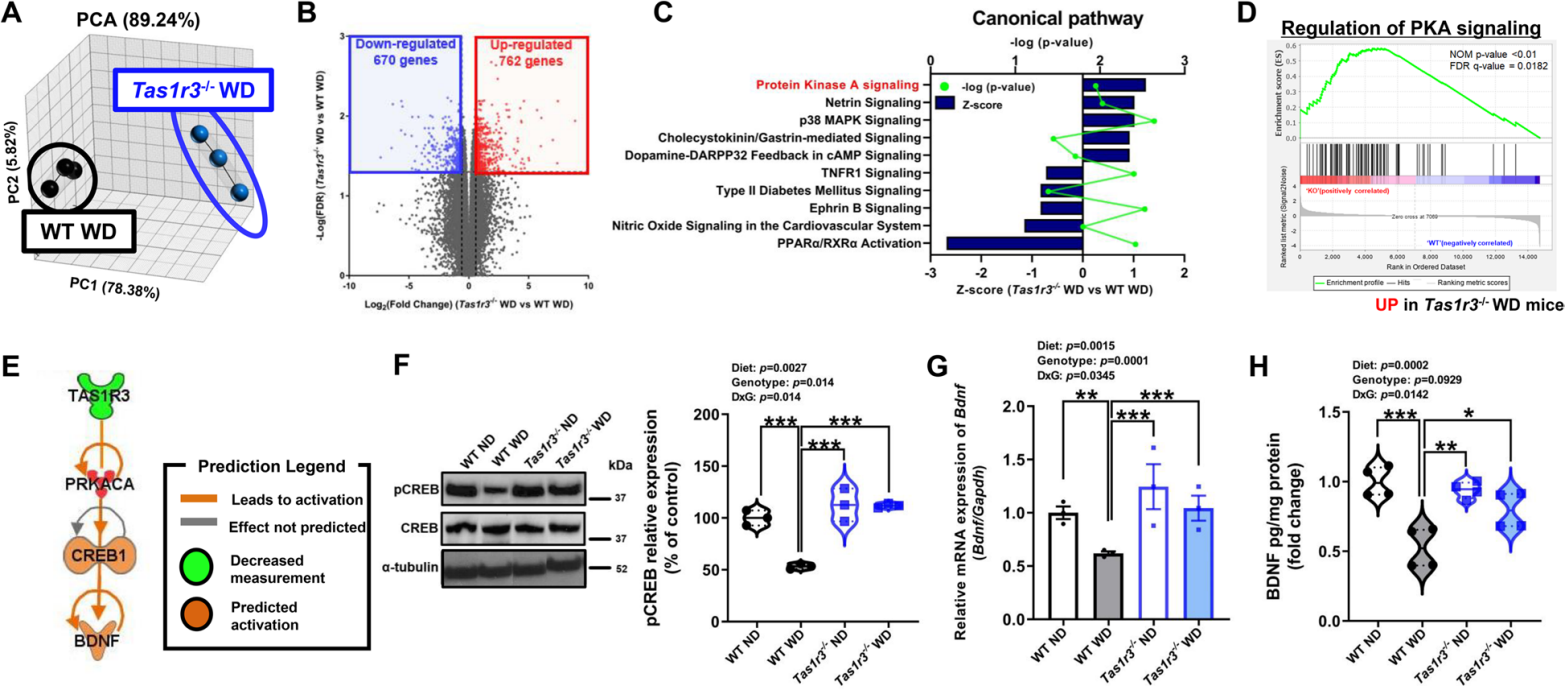
*Tas1r3* deficiency largely alters gene expression in the hypothalamus of WD-fed mice. **A** 3-D view of principal component analysis of the WD-fed *Tas1r3*^−/−^ group versus the WD-fed WT group. Groups are represented by different colors, and dots show individual strains. Black spots represent the WT WD group and blue spots represent the *Tas1r3*^−/−^ WD group. *n* = 3 mice/group. **B** Volcano plot of all DEGs between the WT WD group and the *Tas1r3*^−/−^ WD group. Red dots indicate high relative expression of genes (n = 762), and blue dots indicate low relative expression of genes (n = 670). Unpaired two-tailed Student *t-*test: Thresholds of FDR < 0.05 and 1.5-fold change restriction analysis. **C** Canonical pathway significantly detected in WD-fed *Tas1r3*^−/−^ mice compared to WD-fed WT mice: Right-tailed Fisher’s exact test in IPA. Dots represent –log(p-value) and bars represent Z-score. **D** GSEA related to PKA signaling; genes were ordered by expression fold-changes upon *Tas1r3* deficiency in descending order from left to right. DEGs belonging to the enriched hallmark gene sets identified by GSEA. **E** Transcriptional regulator predicted to regulate DEGs. **F** (Left) Western blot analysis of phosphorylated CREB protein and CREB protein levels in hypothalamus of WT and *Tas1r3*^−/−^ mice fed ND or WD for 12 weeks. (Right) Quantification of the total phosphorylated CREB protein levels in hypothalamus of mice. Two-way ANOVA followed by Tukey’s multiple comparison test: ***p < 0.001. *n* = 3 mice/group. **G** qPCR validation of *Bdnf* mRNA expression*.* Two-way ANOVA followed by Tukey’s multiple comparison test: **p < 0.01, ***p < 0.001. *n* = 3 mice/group. **H** BDNF protein levels in hypothalamus lysates from WT and *Tas1r3*^−/−^ mice fed ND or WD for 12 weeks. Two-way ANOVA followed by Tukey’s multiple comparison test: *p < 0.05, **p < 0.01, ***p < 0.001. *n* = 4 mice/group. All values are presented as mean ± SEM. ANOVA, analysis of variance; BDNF, brain-derived neurotrophic factor; CREB, cAMP response element-binding protein; DEGs, differentially expressed genes; FDR, false discovery rate; GSEA, gene set enrichment analysis; IPA, ingenuity pathway analysis; ND, normal diet; PKA, protein kinas A; WD, western diet; WT, wild-type

Considering that the anxiety levels of WD-fed male WT and *Tas1r3*^-/-^ mice were not explained by their body weight, we investigated the underlying molecular mechanisms in the hypothalamus, a brain structure primarily involved in anxiety. We used RNA sequencing to identify the gene expression profile in the hypothalamus of WD-fed WT mice versus WD-fed *Tas1r3*^-/-^ mice. Principal component analysis revealed that the hypothalamic transcriptome profiles of WD-fed *Tas1r3*^-/-^ mice were distinct from those of WD-fed WT mice (Fig. 3A). We found that 1,432 genes were expressed significantly differently, with 762 upregulated genes and 670 downregulated genes (with a fold change greater than 1.5 or less than -1.5, and a false discovery rate (FDR) <0.05, which were selected as baseline differentially expressed genes (DEGs) in WD-fed *Tas1r3*^-/-^ mice compared to WD-fed WT mice (Fig. 3B).

Next, we investigated the implicit mechanism of WD-induced anxiety prevented by *Tas1r3* deficiency by confirming the top significant canonical pathways by IPA (ingenuity pathway analysis). Protein kinase A (PKA) signaling was the most activated pathway in WD-fed *Tas1r3*^-/-^ mice compared to WD-fed WT mice (*p* = 0.01122; Fig. 3C). Furthermore, gene set enrichment analysis (GSEA) showed that the regulation of PKA signaling was significantly upregulated in WD-fed *Tas1r3*^-/-^ mice compared to WD-fed WT mice (nominal *p* value < 0.01; Fig. 3D).

IPA predicted the activation of CREB (cAMP response element-binding protein) (Fig. 3E), a key downstream target of the PKA signaling cascade, and we confirmed that the phosphorylated CREB protein level was significantly lower in WD-fed WT mice than both ND-fed WT mice and WD-fed *Tas1r3*^-/-^ mice (Fig. 3F). In addition, WD intake decreased *Bdnf* mRNA expression level in WT mice, whereas WD-fed *Tas1r3*^-/-^ mice didn’t show decreased *Bdnf* expression (Fig. 3G). To validate whether differences in the transcript levels of BDNF regulated by *Tas1r3* deficiency, an enzyme-linked immunosorbent assay (ELISA) was conducted. Consistently, the level of hypothalamic BDNF protein was also decreased by WD in WT mice, but not in WD-fed *Tas1r3*^-/-^ mice (Fig. 3H).

CREB and CREB-mediated downstream molecules have been reported to enhance neurogenesis and suppress neuronal cell death [32]. Thus, these results highlight that *Tas1r3* deficiency alters CREB/BDNF signaling in the hypothalamus of WD-fed mice, thereby preventing the pathogenesis of WD-induced anxiety.

### Hypothalamic neurogenesis was maintained in WD-fed *Tas1r3*^*-/-*^ mice compared with WD-fed WT mice

**Fig. 4:**
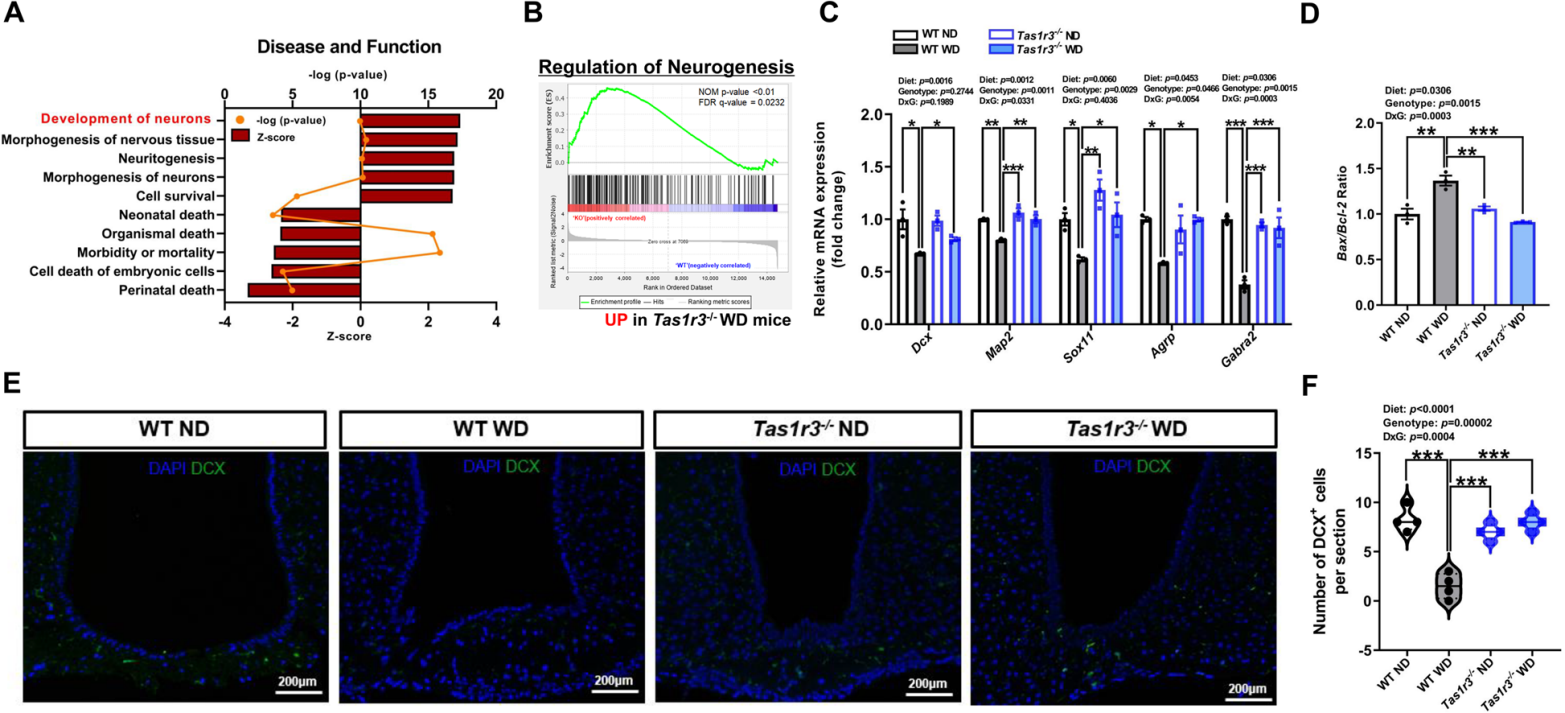
WD-fed *Tas1r3*^−/−^ mice displayed maintained hypothalamic neurogenesis compared to WD-fed WT mice. **A** Significantly detected disease and function in WD-fed *Tas1r3*^−/−^ mice compared to WD-fed WT mice. Right-tailed Fisher’s exact test in IPA. Dots represent –log(p-value) and bars represent Z-score for each disease and function annotated. **B** GSEA related to neurogenesis. **C** qPCR validation of mRNA expression of neuronal progenitor cell markers and hypothalamus-related neuronal cell markers of the hypothalamus. Two-way ANOVA followed by Tukey’s multiple comparison test: *p < 0.05, **p < 0.01, and ***p < 0.001. *n* = 3 mice/group. **D** qPCR validation of *Bax* to *Bcl-2* mRNA expression ratio*.* Two-way ANOVA followed by Tukey’s multiple comparison test: **p < 0.01, ***p < 0.001. *n* = 3 mice/group. **E** DCX staining of regions of the hypothalamus in each group. **F** The number of DCX-positive cells per field in regions of the hypothalamus. Two-way ANOVA followed by Tukey’s multiple comparison test: ***p < 0.001. *n* = 2 slices from 2 mice/group. All data are presented as mean ± SEM. ANOVA, analysis of variance; GSEA, gene set enrichment analysis; IPA, ingenuity pathway analysis; ND, normal diet; WD, western diet; WT, wild-type

To identify the functional annotation of DEGs affected by *Tas1r3*, we categorized these genes based on their relevance to disease and biological functions and tested their Z-scores using IPA. Disease and function analysis revealed that the most increased functions in WD-fed *Tas1r3*^-/-^ mice compared to WD-fed WT mice included the development of neurons (*p* = 1.06E-10), morphogenesis of nervous tissue (*p* = 3.8E-11), and neuritogenesis (*p* = 7.65E-11), whereas the most decreased functions were perinatal death (*p* = 1.07E-05), cell death of embryonic cells (*p* = 5.21E-05), morbidity or mortality (*p* = 1.43E-16) in WD-fed *Tas1r3*^-/-^ mice compared to WD-fed WT mice (Fig. 4A). Furthermore, GSEA confirmed that neurogenesis was significantly upregulated in WD-fed *Tas1r3*^-/-^ mice compared with WD-fed WT mice (nominal *p* value < 0.01; Fig. 4B).

Expectedly, qPCR validation revealed that the transcript levels of *Dcx*, the neuroblast marker, and *Map2*, the mature neuron marker, were markedly decreased in WD-fed WT mice compared to that in ND-fed WT mice. Furthermore, *Agrp* expressed in functional neurons differentiated from hypothalamic neuronal precursor cells and *Gabra2*, a GABAergic neuron marker, were found to be significantly lower than both ND-fed WT mice and WD-fed *Tas1r3*^-/-^ mice (Fig. 4C). In addition, the ratio of *Bax* to *Bcl2*, a marker of cell susceptibility to apoptosis, was significantly increased in WD-fed WT mice relative to ND-fed WT mice whereas it remained significantly decreased in WD-fed *Tas1r3*^-/-^ mice (Fig. 4D).

Nissl bodies are discrete granular structures in neurons that are generally stained to visualize the neurons in the brain. To investigate the quantity of intact neurons in the hypothalamus of mice fed WD, Nissl staining was conducted, and the percentage of intact neurons in selected areas was measured using ImageJ software. We observed a marked decrease in the percentage of intact neuronal cells in hypothalamus sections from WD-fed WT mice compared to both ND-fed WT mice and WD-fed *Tas1r3*^-/-^ mice (see Additional file 1: Fig. S3A-B). Furthermore, the number of DCX-immunopositive cells in WD-fed *Tas1r3*^-/-^ mice was similar to that in ND-fed WT, whereas it decreased in WD-fed WT mice compared to that in ND-fed WT mice (Fig. 4E-F).

These results suggest that *Tas1r3* deficiency alleviates the WD-induced suppression of neurogenesis in the hypothalamus.

### *Tas1r3* knockdown prevented WD-induced suppression of CREB/BDNF signaling in the adult hypothalamic neuronal cell line

**Fig. 5:**
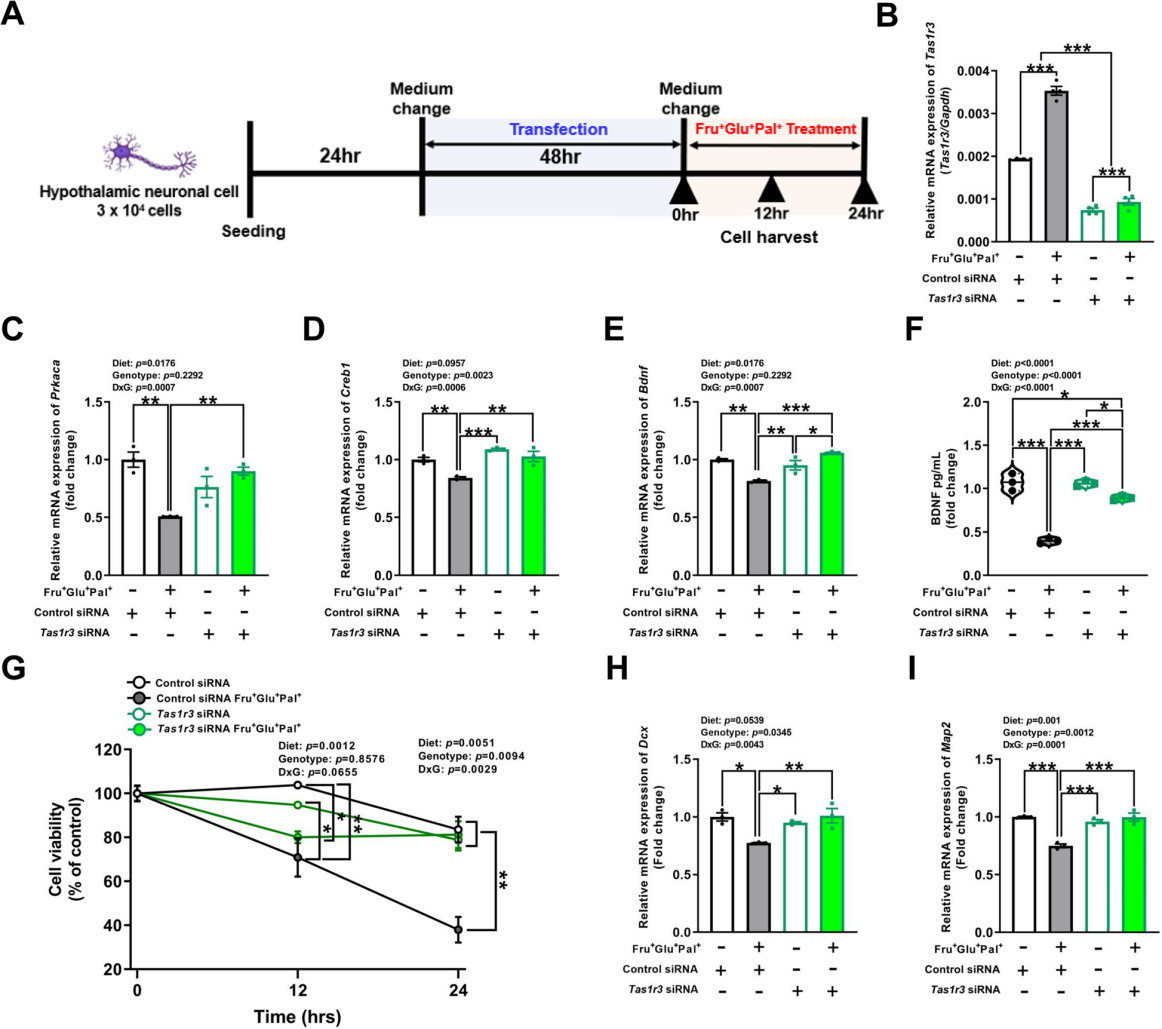
*Tas1r3* knockdown alleviates high glucose, fructose and palmitate-induced suppression of CREB/BDNF signaling in hypothalamic neuronal cell. **A** Study design of siRNA knockdown in hypothalamic neuronal cell line mHypoA-2/12. **B** Evaluation of siRNA-mediated *Tas1r3* mRNA suppression in a cultured adult hypothalamic neuronal cell line after 48 h of transfection. Two-way ANOVA followed by Tukey’s multiple comparison test: ***p < 0.001. *n* = 4/group. **C-E** mHypoA-2/12 cells were transfected with *Tas1r3* siRNA or scrambled control siRNA for 48 h and stimulated with or without fructose (10 mM), glucose (10 mM), and palmitate (10 μM) for 24 h. Relative mRNA expression of *Prkaca*, *Creb1*, and *Bdnf.* Two-way ANOVA followed by Tukey’s multiple comparison test: *p < 0.05, **p < 0.01, and ***p < 0.001. *n* = 3/group. **F** Protein levels of BDNF released into the supernatant. Two-way ANOVA followed by Tukey’s multiple comparison test: **p < 0.01, ***p < 0.001. *n* = 3/group. **G** mHypoA-2/12 cell viability at 0, 12, and 24 h. Two-way ANOVA followed by Tukey’s multiple comparison test: *p < 0.05, **p < 0.01. *n* = 3/group. **H,I** Relative mRNA expression of *Map2* and *Dcx*. Two-way ANOVA followed by Tukey’s multiple comparison test: *p < 0.05, **p < 0.01, and ***p < 0.001.* n* = 3/group. All data are presented as mean ± SEM. ANOVA, analysis of variance; BDNF, brain-derived neurotrophic factor; CREB, cAMP response element-binding protein

Since we used conventional knockout mice, we investigated whether *Tas1r3* directly modulated CREB/BDNF signaling in hypothalamic neurons. We transfected the hypothalamic neuronal cell line, mHypoA-2/12 cells with siRNA targeting *Tas1r3* and treated the cell culture media containing 10mM fructose, 10mM glucose, and 10μM palmitate (a major component of dietary fat) to create WD conditions for the cell line (Fig. 5A). We found that *Tas1r3* mRNA expression was significantly suppressed by siRNA compared to the control. Furthermore, we confirmed that the WD condition significantly increased *Tas1r3* expression (Fig. 5B). The knockdown of *Tas1r3* significantly prevented the decrease in the mRNA expression of *Prkaca*, *Creb1,* and *Bdnf* induced by the WD condition (Fig. 5C-E). ELISA-based quantification of BDNF protein released into the supernatant validated the changes in the transcript levels of *Bdnf* by *Tas1r3* knockdown (Fig. 5F). Furthermore, cell viability calculated at 12 hr after ND- or WD-conditioned treatment, both groups conditioned with WD showed lower cell viability compared to controls. However, decrease in viability at 24 hr was not observed in the WD-conditioned *Tas1r3* siRNA group, whereas WD-conditioned control siRNA group showed significantly lower viability compared to both the control and WD-conditioned *Tas1r3* siRNA group (Fig. 5G). As expected, the *Tas1r3* knockdown ameliorated suppressed expression of *Dcx* and *Map2* by WD conditioned media (Fig. 5H-I).

## Discussion

We demonstrated that prolonged WD consumption can exacerbate anxiety in male mice, independent of weight gain, as reported in previous studies [33–35]. Furthermore, TAS1R3 modulates CREB-mediated neuronal regeneration in WD-fed mice. Ultimately, we displayed that TAS1R3 is a key regulator of nutritional stimulus-induced anxiety by altering hypothalamic function.

Obesity, which is caused by long-term high-calorie consumption, can increase anxiety through insulin or leptin resistance, systemic inflammation, and hormonal dysregulation [36]. TAS1R3, which is expressed in metabolic tissues such as adipocytes and pancreatic β cells, has been reported to regulate metabolism [37, 38]. Studies on mice with genetic ablation of TAS1R3 revealed differences in weight gain compared to WT mice [39, 40]. Here, in our *in vivo* model, WD-fed *Tasr3*^*-/-*^ mice also showed less weight gain compared to WD-fed WT mice. However, we confirmed that the anxiety levels of mice were independent of their body weights by linear regression analysis and ANCOVA. These results suggest that molecular mechanisms in the brain, not weight gain, may be involved in the alterations of anxiety-related phenotypes caused by *Tas1r3* deficiency.

Regarding TAS1R3 and brain functions, only behavioral dysfunction by hippocampal neurodegeneration in *Tas1r3* deficient mice fed a normal chow diet have previously been reported [41]. In contrast, in our model, in which *Tas1r3* deficient mice were fed WD for 12 weeks, *Tas1r3* deficiency-mediated neuroprotective effects in the hypothalamus of WD-fed mice reduced WD-induced immobility and anxiety-like behaviors, but there were no behavioral changes in ND-fed mice. Unlike the hypothalamus, the WD condition did not alter *Tas1r3* mRNA expression levels in the hippocampus and hippocampal neuronal cell line (see Additional file 1: Fig.S5A-B). Furthermore, there was no significant difference in *Prkaca*, *Creb1* and *Bdnf* mRNA expression in hippocampus and hippocampal neuronal cell line between groups (see Additional file 1: Fig. S5C-D). These results suggest that TAS1R3 may have different regulatory activities, depending on the brain region and nutritional status.

The molecular mechanisms by which TAS1R3 regulates anxiety remain unclear. Hence, we performed transcriptomic analysis of the hypothalamus of WD-fed WT and *Tas1r3*^-/-^ mice. PKA signaling was identified as the main pathway that was increased in WD-fed *Tas1r3*^-/-^ mice compared to WD-fed WT mice. The hypothalamus is characterized as the center of integration of peripheral nutrient-related signals, such as circulating adiposity hormones, gastric hormones, and nutrients [42]. Particularly, the median eminence of the hypothalamus is in contact with fenestrated capillaries, providing privileged access to nutritional signals carried by the blood stream [25]. Furthermore, the PKA signaling cascade in the hypothalamus can be reportedly activated by nutritional signals, such as fasting state, or suppressed by a hypercaloric diet by G-protein coupled receptor-mediated mechanisms signaling [27, 43], suggesting a potential mechanism by which interruption of nutrient sensing by TAS1R3 prevents WD-induced suppression of PKA.

In the brain, the interplay between the transcriptional controller CREB, a key downstream target of PKA, and neurotrophic ligands such as brain-derived neurotrophic factor (BDNF) is crucial for synaptic plasticity and neuronal development [44, 45]. Compared to WD-fed WT mice, the hypothalamus of WD-fed *Tas1r3*^-/-^ mice showed significant increases in the phosphorylated CREB protein and CREB-mediated genes such as *Arc, Erg1, Vegfa,* and *Bdnf.* CREB is activated by multiple signaling pathways during synaptic strengthening, plasticity, and neurogenesis, which are related to brain function [46, 47]. Furthermore, BDNF levels were found to be lower in people with anxiety disorders [48] and hypothalamic gene transfer of BDNF reduced anxiety-like behaviors in rodents [49, 50]. BDNF can also induce hypothalamic neurogenesis, which can affect diverse functions of the hypothalamus [51].

Although hippocampal neurogenesis has received the most attention [52], evidence of hypothalamic neurogenesis and its importance in biological functions has recently been reported [53]. Here, we found that neuronal development was the most activated biological function in WD-fed *Tas1r3*^-/-^ mice compared to WT mice. Furthermore, chronic consumption of a high-calorie diet suppressed neuronal turnover in the arcuate nucleus by increasing apoptosis of newborn neurons [54]. We found that the ratio of *Bax* to *Bcl2* (a marker of cell susceptibility to apoptosis) [55], was significantly decreased in WD-fed *Tas1r3*^-/-^ mice, suggesting that *Tas1r3* deficiency may increase resistance to WD-induced neuronal death. Anxiety is affected by hypothalamic neurogenesis by modulating the differentiation of anxiolytic neurons [56]. Neuronal stem cells in the hypothalamus differentiate into functional neurons that express the neuropeptides AgRP and POMC, which are involved in feeding regulation [57]. Interestingly, we confirmed that the mRNA expression level of *Agrp* was increased in WD-fed *Tas1r3*^-/-^ mice compared to WD-fed WT mice. AgRP neurons have been reported to modulate fasting-induced anxiolytic effects [58], suggesting that decreased AgRP-expressing neurons in WD-fed WT mice might increase anxiety-like phenotypes. Moreover, *Gabra2*, a GABAergic neuron marker, was significantly increased in WD-fed *Tas1r3*^-/-^ mice compared to WT mice. The GABA_A_α2 subunit (the *Gabra2* gene) plays an important role in the regulation of anxiety being modulated by benzodiazepines, barbiturates, and ethanol [59, 60]. These results indicate that enhanced hypothalamic neurogenesis in WD-fed *Tas1r3*^-/-^ mice compared to WD-fed WT mice may lead to the regeneration of anxiety-regulating neurons.

To the best of our knowledge, this is the first report to identify a key regulator of neuronal function in the hypothalamus during stress induced by diet, one of the key environmental factors in our lives. The findings from our rodent model confirmed that *Tas1r3* deficiency prevents WD-induced neuronal loss and anxiety. However, despite the genetic similarity, further human studies are needed to investigate the association between TAS1R3 expression levels and anxiety status in people consuming Western diets.

## Conclusions

In summary, we demonstrated that *Tas1r3* deficiency maintains neuronal regeneration in the hypothalamus of male mice under dietary stimuli by activating CREB/BDNF signaling, which regulates neuronal plasticity and development. A key finding of our study is that nutrient molecules, rather than peripheral hormones, directly affect neuronal survival and renewal via the nutrient-sensing receptor, TAS1R3. Additionally, TAS1R3 is a GPCR, which is a major therapeutic target for approved drugs [61], suggesting that it could be a novel therapeutic target for diet-induced anxiety. The present study provides the first comprehensive description of the roles of TAS1R3 in the hypothalamus, providing additional evidence of causality between WD and anxiety and revealing the previously unexplained mechanism of WD-induced anxiety.

## Methods

### Mice and diets

WT C57BL/6 (RRID:IMSR_JAX 000664) and Tas1r3tmCSZ (RRID:IMSR_JAX 013066) mice were purchased from Jackson Laboratory (Bar Harbor, ME, USA). Male heterozygous with female heterozygous mice were used to generate *Tas1r3*^-/-^ and littermate WT mice. *Tas1r3*^-/-^ mice were backcrossed with WT C57BL/6 mice for at least seven generations. *Tas1r3*^-/-^ mice weighing 23–25 g and age- and weight-matched WT littermates were housed in a specific pathogen-free animal facility. A high-fat diet (D12492) and matched control diet (D12450J) were purchased from Research Diet Inc. (New Brunswick, NJ, USA). Male WT and *Tas1r3*^-/-^ mice were randomly divided into four groups: (1) WT ND, WT mice received a control diet with plain water ; (2) *Tas1r3*^-/-^ ND, *Tas1r3*^-/-^ mice received a control diet with plain water; (3) WT WD, WT mice received a high-fat diet with 30% (w/v) sucrose solution; and (4) *Tas1r3*^-/-^ WD, *Tas1r3*^-/-^ mice received a high-fat diet with 30% (w/v) sucrose solution. Food and drinks were provided *ad libitum* for 12 weeks. Mice were group-housed with 2–3 mice per cage in the same room to minimize any environmental effects and maintained according to the guidelines of the Seoul National University Animal Experiment Ethics Committee. All experiments were approved by the Institutional Animal Care and Use Committee of Seoul National University (approval number: SNU-181001-2; Seoul, Korea).

### Behavioral assessment

Two cohorts of animals were produced (Additional file 2: Table S1) to conduct behavioral testing. Behavioral tests were conducted to assess locomotor activities and anxiety-like behaviors as detailed below.

#### Open field test

Locomotor activity and anxiety-like behaviors of the mice were assessed using a rectangular chamber (W × L × D = 40 cm × 40 cm × 40 cm). The mice were acclimatized to the room for 1h before the test. The mice were then introduced into the chamber, and all mouse activities were recorded for 10 min. Activity-monitoring software (ANY-Maze, Stoelting Co., Wood Dale, IL, USA) was used to calculate multiple parameters, such as total distance traveled, velocity, and mobility over the duration of the 10-min test. Freezing time in the central 25% of the chamber (center zone) and entries or line crossings to the center zone were measured to quantify anxiety. At the end of each test, the chamber was cleaned using 70% ethanol.

#### Elevated plus maze test

Anxiety-related behaviors were assessed using an EPM. The entire apparatus was 50 cm above the floor and consisted of two open arms (W × L = 5 cm × 30 cm) and two closed arms (W × L = 5 cm × 30 cm) attached to a central platform (W × L = 5 cm × 5 cm). Mice were placed on the center platform of the apparatus facing an open arm, and mouse movements were recorded for 5 min. ANY-Maze software (Stoelting Co., Wood Dale, IL, USA) was used to calculate the time spent in the arms, to quantify anxiety. The time spent in the closed or open arms was divided by the total time spent in the arms. At the end of each test, the apparatus was cleaned using 70% ethanol.

#### Light-Dark box test

Anxiety-related behaviors were assessed using the LDB test. The LDB test was conducted as described previously [41]. A dark chamber (W × L × D = 40 cm × 20 cm × 40 cm) was inserted to the rectangular chamber used in the open field test to perform this test. The LDB was divided into a dark (lux 1) and a light (lux 400) zone and interconnected by an entry (W × L = 7 cm × 7 cm) in the partition wall. Mice were placed on the center of the dark zone and mouse movements were recorded for 10 min. Anxiety was calculated based on the time spent in the light zone using ANY-Maze software (Stoelting Co., Wood Dale, IL, USA). At the end of each test, the chamber was cleaned using 70% ethanol.

#### Novelty-suppressed feeding test

To assess anxiety behavior, NSF test was conducted. Food was removed at 24 hr before testing, although water was available *ad libitum*. For testing, mice were placed in an open field (W × L × D = 50 cm × 50 cm × 20 cm) that contained a food pellet on a circular filter paper (10 cm in diameter) in the center of the apparatus. Mice were monitored for 5min and the latency to approach the food pellet was measured to quantify anxiety.

### RNA isolation and gene expression profiling by RNA-seq

After 12 weeks of diet induction, all mice were anesthetized and euthanized by intraperitoneal injection of 20% urethane. Tissue samples from the hypothalamus stored at -80 °C were homogenized using TissueLyser II (QIACEN, Crawley, UK), and total RNA was extracted and purified using a DNA-free RNA isolation kit (RNAqueous-4PCR kit; Ambion, Austin, TX, USA), according to the manufacturer’s instructions. Before RNA sequencing, the quality and concentration of extracted total RNA was confirmed using an Agilent 2100 Bioanalyzer (Agilent Technologies, Palo Alto, CA, USA). Out of the samples with an RNA integrity number greater than 8 were randomly selected for sequencing. *n* = 3 mice/group.

### Bioinformatic analysis of Next-Generation RNA-seq

Raw RNA-seq reads were divided into individual samples based on their barcodes, and quality was controlled using the FASTQC tool as previously described [62]. The reads were analyzed using Partek Flow software (Parteck, St. Louis, MO, USA; http://www.partek.com/partekgs). Briefly, the reads were annotated to the Genome Reference Consortium Mouse Build 39 (mm39) using the STAR method. Quantification was conducted using RefSeq release 93. Differential expression analysis was performed using the R package DESeq2. Genes showing an absolute 1.5-fold-change and FDR <0.05 were used as DEGs. DEGs were subjected to ingenuity pathway analysis using ingenuity pathway analysis (IPA) software (www.ingenuity.com). The statistical significance of the enrichment of specific pathways among the DEGs was investigated using Fisher’s exact test. Our RNA-seq data have been submitted to the Gene Expression Omnibus (accession number GSE216063).

### Protein extraction and western blotting

Hypothalamus isolated from mice was transferred to 300μl Tissue Extraction Reagent 1 (Cat# FNN0071; Invitrogen) with 3μl protease inhibitor (Cat# P-2714; Sigma-Aldrich, St. Louis, MO, USA) and homogenized. Pierce BCA Protein Assay Kit (Cat# 23227; Thermo Fisher Scientific) was used to determine the conentration of protein samples. Each protein lysate (20μg) was run on sodium dodecyl sulfate polyacrylamide gel electrophoresis gel, transferred to a polyvinylidene fluoride membrane, blocked for 1 h at 24 °C with 5% fat-free milk in Tris-buffered saline containing 0.1% Tween 20, and incubated with monoclonal rabbit anti-CREB (1:500) (Cat# ab32515; Abcam, Cambridge, UK; RRID:AB_2292301), rabbit and anti-phospho-CREB (1:1,000) (Cat# 9198S; Cell Signaling Technology, Danvers, MA, USA; RRID:AB_2561044). Mouse anti-α-tubulin (1:10,000) (Cat# T5168, Sigma-Aldrich, St. Louis, MO, USA; RRID:AB_477579) was used as a control. Horseradish peroxidase-conjugated goat anti-rabbit secondary antibodies (1:5,000) (Cat# 7074S; Cell Signaling Technology, Danvers, MA, USA; RRID:AB_2099233) and goat anti-mouse secondary antibodies (1:10,000) (Cat# G21040; Invitrogen, Carlsbad, CA, USA; RRID:AB_2536527) were used for detection. Enhanced chemiluminescence western blot detection reagents (Amersham Pharmacia Biotech, Piscataway, NJ, USA) were used to detect the target proteins. *n* = 3 mice/group.

### Enzyme-linked immunosorbent assay

BDNF protein expression was quantified using a duo-set ELISA assay (Cat# DY248, R&D systems, Minneapolis, MN, USA) according to the manufacture’s instructions. Briefly, standards and samples diluted with assay buffer were incubated with detection antibody for 2 hrs at room temperature. TMB was used as the substrate to detect the bound detection antibodies. Optical density was measured at 450nm. *n* = 4 mice/group.

### Nissl staining

After the 12^th^ week of diet consumption, transcardial perfusion of the mice was performed with 4% paraformaldehyde for 14-16h fixation. The tissues were processed and embedded in paraffin. A rotary microtome was used to section the hypothalamus. The 4 μm-thick hypothalamic coronal sections were deparaffinized and hydrated using distilled water. Cresyl violet acetate (1.0% aqueous) was applied to the tissue for 5 min and then quickly rinsed in 1 mL of distilled water. After quick dehydration in absolute alcohol, the tissues were cleared in xylene and mounted in synthetic resin. Motic Digital Slide Assistant software v1.0.7.61b (Motic China Group Co., Ltd, NY, USA) was used to analyze the histology of the samples. To count Nissl-stained cells in the hypothalamus, ImageJ software v1.53e (National Institutes of Health, Bethesda, MD, USA) was used. *n* = 4 slices from 2 mice/group.

### Immunohistochemistry

Hypothalamus sections of 4 μm thickness were prepared from paraffin-embedded specimens. To identify newborn neurons (DCX^+^), we conducted dewaxing, antigen retrieval, antibody incubation, staining, and sealing. DCX antigen was retrieved using sodium citrate (pH 6∙0). Sections were incubated 14-16h at 4 °C with the primary antibody anti-DCX (1:200; Cat# ab18723; Abcam). The sections were then incubated with goat anti-rabbit IgG (Cat# A16101, ThermoFisher Scientific, USA). Immunoreactive cells were visualized and captured using a fluorescence microscope (BX53, Olympus, Tokyo, Japan) and digital camera (INFINITY3, Lumenera, Ottawa, Canada) at 200 × magnification. ImageJ software v1.53e (National Institutes of Health, Bethesda, MD, USA) was used for analysis. *n* = 2 slices from 2 mice/group.

### Cell culture

mHypoA-2/12 cells (Cat # CLU177; Cellutions Biosystem Inc., Toronto, Canada) were cultured in 1 × Dulbecco’s modified Eagle’s medium (Cat # LM001-07; WelGENE Inc., Daegu, Korea) supplemented with 10% fetal bovine serum and 1% penicillin/streptomycin at 37 °C with 5% CO_2_.

### In vitro knockdown with siRNA

For the knockdown of *Tas1r3*, 3x10^4^ mHypoA-2/12 cells per well were seeded in a 6-well culture plate approximately 24 hr prior to transfection. After the cells had grown to 50–60% confluency, 40nM of *Tas1r3* siRNA (Bioneer, Daejeon, Korea) and negative control siRNA (Bioneer) complexed with Lipofectamine RNAiMAX® (Invitrogen, ThermoFisher Scientific, USA) were added to each well according to the manufacturer’s instructions and incubated for 48 hr at 37 °C. On the day of the experiments, the transfection medium was removed, and the supernatants were replaced with no glucose medium (Cat # LM001-06, WelGENE) or medium containing 10mM glucose, 10mM fructose and 10μM palmitate. The cells were then incubated for 0, 12, and 24 hr at 37 °C. Following incubation, cell viability at each time point was measured using Countess^TM^ 3 Automated Cell Counters (Invitrogen). Then, the cells were washed with phosphate buffered saline, and RNA was isolated for qPCR. The target sequences of the siRNA are listed in Additional file 2: Table S2.

### Quantitative real-time PCR (qPCR)

Reverse transcription and real-time PCR were conducted as previously described [63]. The primers used for qPCR are listed in Additional file 2: Table S3. *n* = 3/group.

### Statistical analyses

Two-way ANOVA was used for the statistical analysis of multiple experimental groups with a Tukey’s post-hoc test. Analysis of anxiety-related parameters with body weight as the covariate was performed using ANCOVA. An unpaired two-tailed Student’s *t*-test was used to compare two groups. Differences were considered significant when the *p*-value was < 0.05. All data are presented as mean ± SEM. In gene expression profiling, a right-tailed Fisher’s exact test was used to determine the significant biological pathways enriched with DEGs. GraphPad Prism version 9.00 for Windows (GraphPad, Inc., San Diego, CA, USA), IBM SPSS Statistics v25 (IBM Corp., Armonk, NY, USA), Partek Flow software (Partek, St. Louis, MO, USA; http://www.partek.com/partekgs), and IPA were used for the statistical analyses.

### Supplementary Information

**Additional file 1: Fig. S1.** Caloric intake of male WT and *Tas1r3*^−/−^ mice fed ND or WD for 12 weeks. **Fig. S2.** Correlation graphs between body weight and anxiety-related parameters. **Fig. S3.** Body weight, locomotor activities and anxiety-related behaviors of female WT and *Tas1r3*^−/−^ mice fed ND or WD for 12 weeks. **Fig. S4.** Representative images and quantitative analysis of Nissl staining of hypothalamus. **Fig. S5.** *Tas1r3*, *Prkaca*, *Creb1* and *Bdnf* mRNA expression in the hippocampal tissue and cultured adult hippocampal neuronal cell line.

**Additional file 2: Table S1.** Cohorts of animals used for behavioral testing. **Table S2.** siRNA target sequences. **Table S3. **qPCR primers.

**Additional file 3: Raw Data.** Raw data underlying Fig. 1, Figs. 3-5, and Fig. S5.

**Additional file 4: Fig. S6.** Uncropped western blot figures.

## Data Availability

All data generated or analysed during this study are included in this published article, its supplementary information files and publicly available repositories. The raw transcriptome datasets generated during the current study are available in the Gene Expression Omnibus database (available online: http://www.ncbi.nlm.nih.gov/geo) (accession numbers GSE216063) The data that support the findings of this study are available from the corresponding author upon reasonable request. The individual data values for Figs. 1, 3, 4, and 5, as well as Additional file 1: Fig. S5, are provided in Additional file 3.

## Abbreviations

ANCOVA: One-way analysis of covariance
ANOVA: Analysis of variance
BDNF: Brain-derived neurotrophic factor
CREB: CAMP response element binding protein
DEGs: Differentially expressed genes
ELISA: Enzyme-linked immunosorbent assay
EPM: Elevated plus maze
FDR: False discovery rate
GSEA: Gene set enrichment analysis
IPA: Ingenuity pathway analysis
LDB: Light–dark box
ND: Normal diet
NSF: Novelty-suppressed feeding
OF: Open field
PKA: Protein kinase A
TAS1R3: Taste receptor type 1 member 3
WD: Western diet
WT: Wild-type
