# Supplementary material for: Taste receptor type 1 member 3 enables western diet-induced anxiety in mice
Source: BMC Biol. 2023 Nov 6;21:243. doi: 10.1186/s12915-023-01723-x (PMC10626698; doi:10.1186/s12915-023-01723-x)
Supplement: Supplementary file 1 — Additional file 1: Fig. S1. Caloric intake of male WT and Tas1r3−/− mice fed ND or WD for 12 weeks. Fig. S2. Correlation graphs between body weight and anxiety-related parameters. Fig. S3. Body weight, locomotor activities and anxiety-related behaviors of female WT and Tas1r3−/− mice fed ND or WD for 12 weeks. Fig. S4. Representative images and quantitative analysis of Nissl staining of hypothalamus. Fig. S5. Tas1r3, Prkaca, Creb1 and Bdnf mRNA expression in the hippocampal tissue and cultured adult hippocampal neuronal cell line. [file 12915_2023_1723_MOESM1_ESM.pdf]

Figure S1

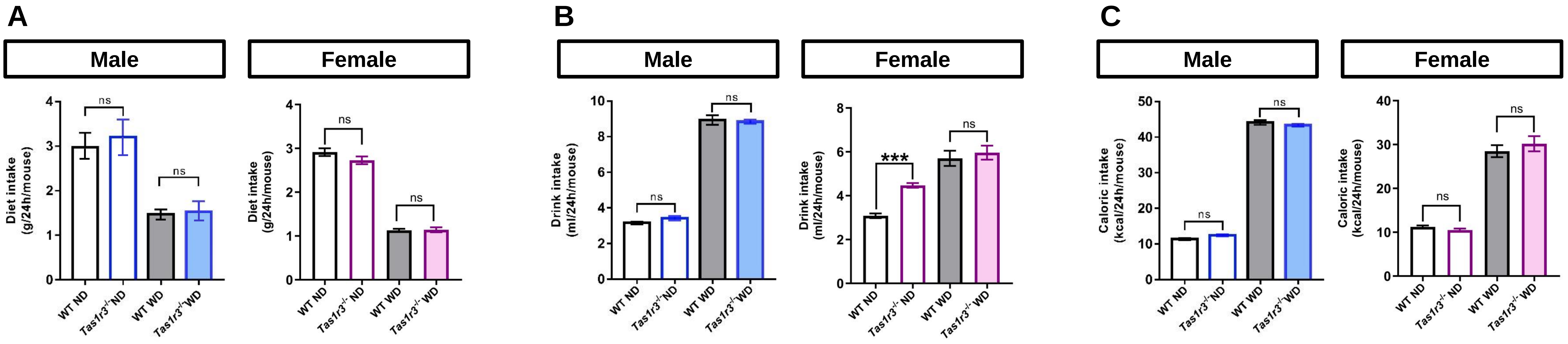

Additional file 1: Fig. S1. Caloric intake of male WT and *Tas1r3*$^{-/-}$ mice fed ND or WD for 12 weeks.

(A) Diet intake, (B) Drink intake, and (C) Caloric intake in male and female WT and *Tas1r3*$^{-/-}$ mice fed ND or WD. Unpaired two-tailed Student *t*-test. *n* = 10/group. All values are presented as the means ± SEM. ND, normal diet; ns, not significant; WD, western diet; WT, wild-type.

Figure S2

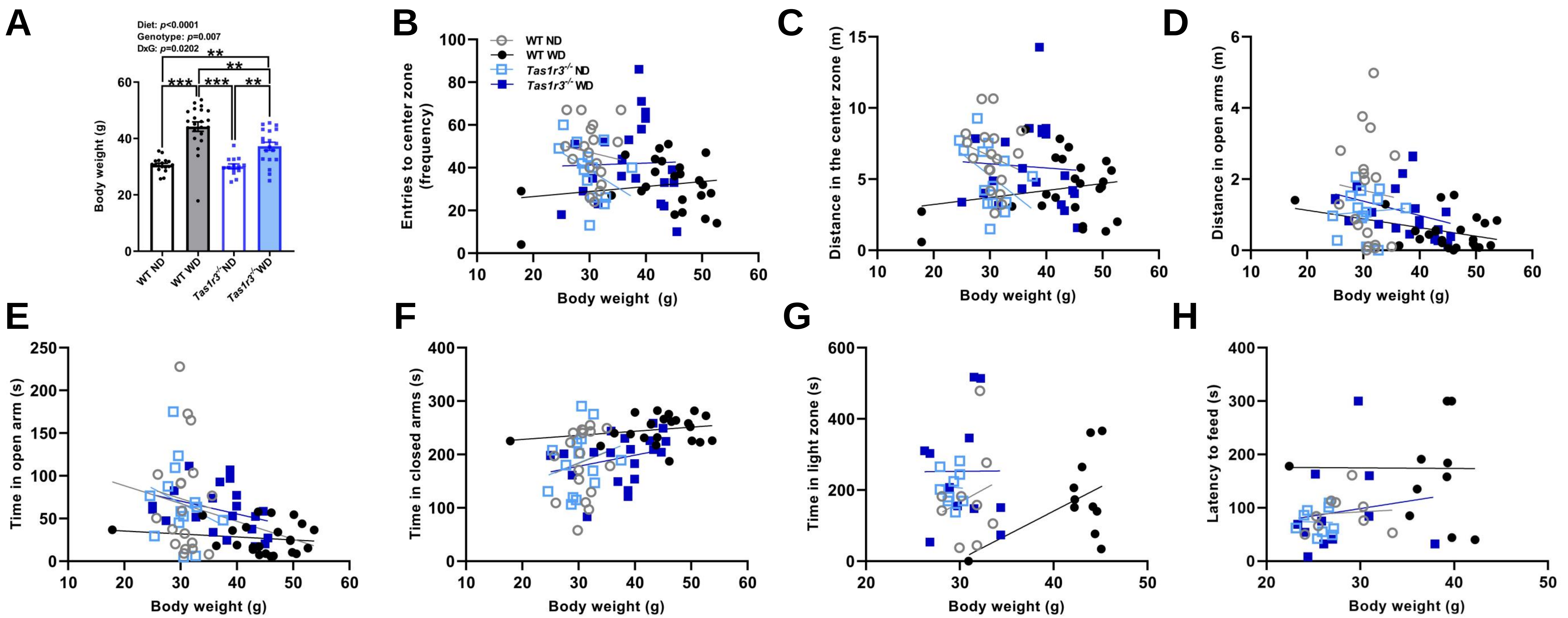

Additional file 1: Fig. S2. Correlation graphs between body weight and anxiety-related parameters.

(A) Body weight of male WT and *Tas1r3*$^{-/-}$ mice fed ND or WD. Two-way ANOVA followed by Tukey's multiple comparison test: *p<0.05, **p<0.01, ***p<0.001. All values are presented as the means ± SEM. No significant correlations (linear regression) were found in each group between body weight and anxiety-related parameters: (B) frequency of entries to center zone (WT ND, r^2 = 0.01478, p = 0.6309; WT WD, r^2 = 0.03091, p = 0.4112; KO ND, r^2 = 0.1663, p = 0.1477; KO WD, r^2 = 0.0009577, p = 0.8999), (C) distance in the center zone (WT ND, r^2 = 0.04608, p = 0.3923; WT WD, r^2 = 0.04264, p = 0.333; KO ND, r^2 = 0.1962, p = 0.1127; KO WD, r^2 = 0.04608 p = 0.3923), (D) distance in open arms (WT ND, r^2 = 0.003772, p = 0.8087; WT WD, r^2 = 0.1434, p = 0.0681; KO ND, r^2 = 0.005174, p = 0.8070; KO WD, r^2 = 0.122, p = 0.1206), (E) time spent in open arms (WT ND, r^2 = 0.006795, p = 0.7450; WT WD, r^2 = 0.02278, p = 0.4814 KO ND, r^2 = 0.06018, p = 0.3979; KO WD, r^2 = 0.1208, p = 0.1226), (F) time spent in closed arms (WT ND, r^2 = 0.02905, p = 0.499; WT WD, r^2 = 0.05417, p = 0.2737; KO ND, r^2 = 0.05744, p = 0.4092; KO WD, r^2 =0.0902, p = 0.1859), (G) time spent in light zone (WT ND, r^2 = 0.04530, p = 0.5825; WT WD, r^2 = 0.2084, p = 0.1581; KO ND, r^2 = 0.002286, p = 0.9028; KO WD, r^2 = 2.2E-05, p = 0.9891), (H) latency to feed (WT ND, r^2 = 0.006582, p = 0.8356; WT WD, r^2 = 3.496E-05, p = 0.9862; KO ND, r^2 = 0.0005621, p = 0.9517; KO WD, r^2 = 0.01929, p = 0.6838). WT ND: n = 18; WT WD: n = 24; *Tas1r3*$^{-/-}$ ND: n = 14; *Tas1r3*$^{-/-}$ WD: n = 21. ANOVA, analysis of variance; ND, normal diet; WD, western diet; WT, wild-type.

Figure S3

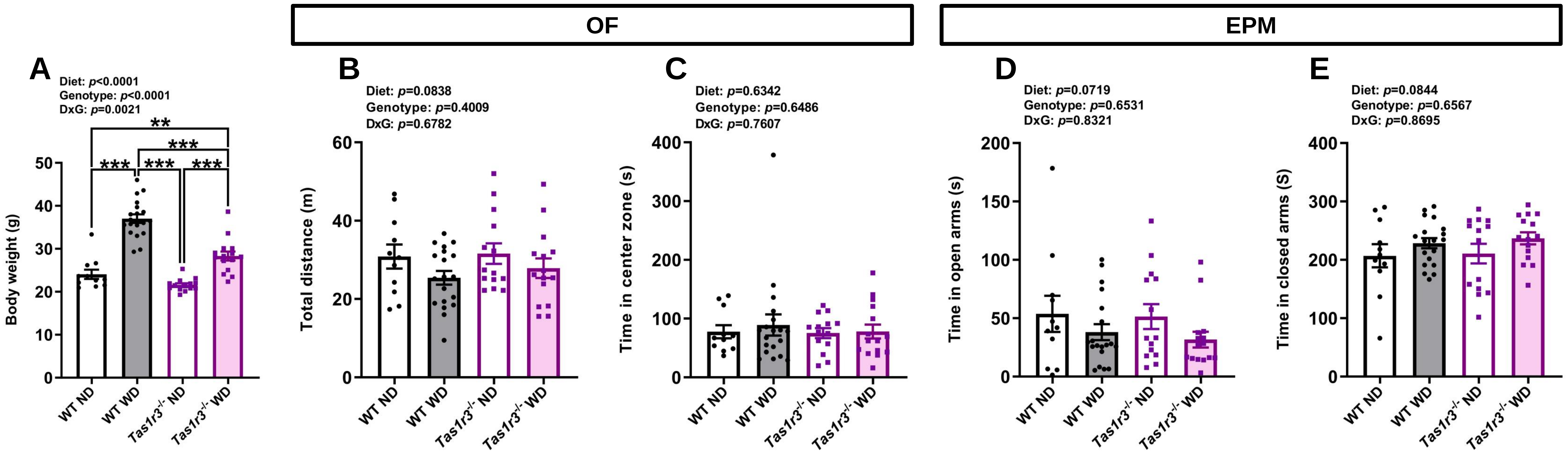

Additional file 1: Fig. S3. Body weight, locomotor activities and anxiety-related behaviors of female WT and *Tas1r3*$^{-/-}$ mice fed ND or WD for 12 weeks.

(A) Body weight of female WT and *Tas1r3*$^{-/-}$ mice fed ND or WD for 12 weeks. Two-way ANOVA followed by Tukey's multiple comparison test: ***p*<0.01, ****p*<0.001. (B) Locomotor activities and (C-E) anxiety related behaviors measured in OF and EPM test. Two-way ANOVA followed by Tukey's multiple comparison test. WT ND: *n* = 11; WT WD: *n* = 19; *Tas1r3*$^{-/-}$ ND: *n* = 14; *Tas1r3*$^{-/-}$ WD: *n* = 15. All values are presented as the means ± SEM. ANOVA, analysis of variance; EPM, elevated plus maze; ND, normal diet; OF, open field; WD, western diet; WT, wild-type.

Figure S4

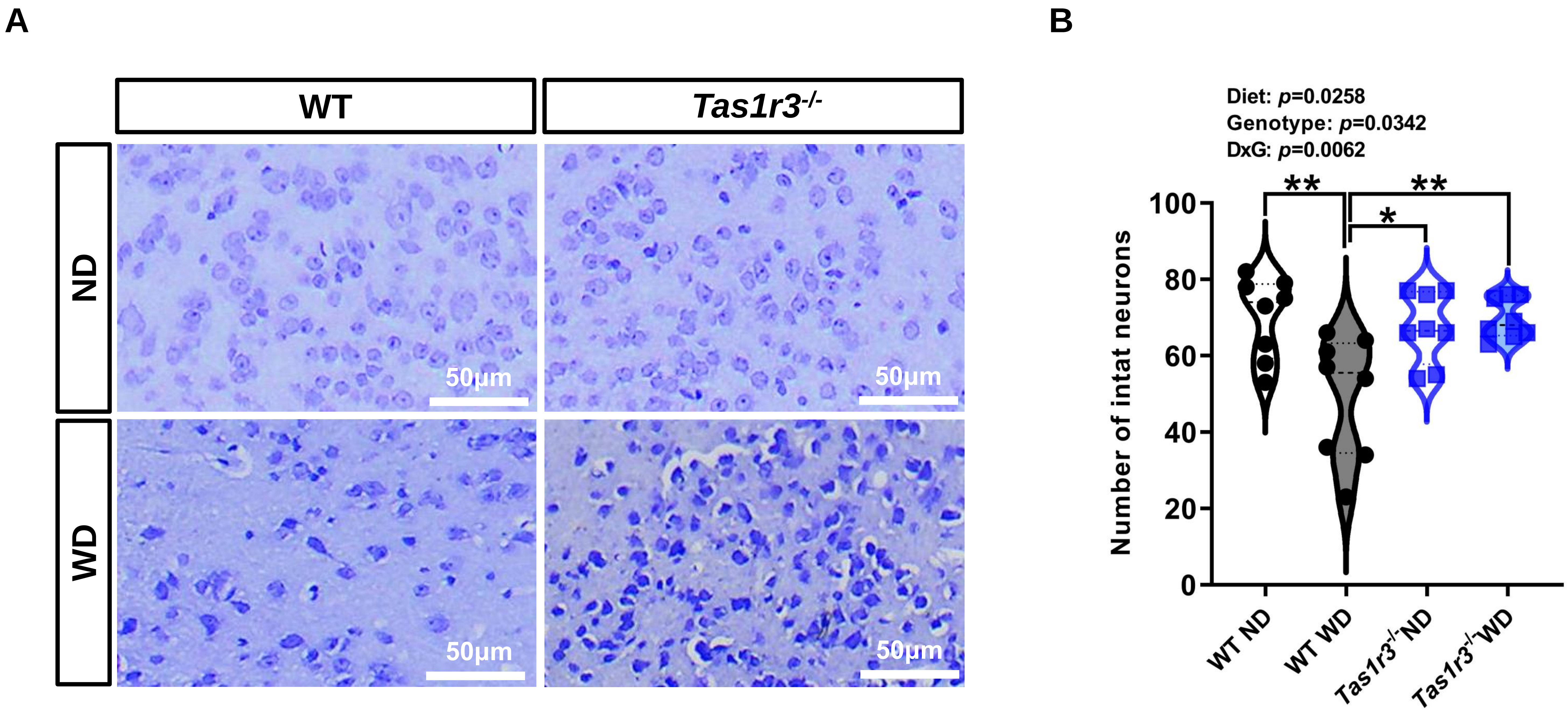

Additional file 1: Fig. S4. Representative images and quantitative analysis of Nissl staining of hypothalamus.

(A) Representative sections of the Nissl staining of hypothalamus. (B) The number of intact neurons per field in regions of hypothalamus. Two-way ANOVA followed by Tukey's multiple comparison test: *p<0.05, **p<0.01. n = 4 slices from 2 mice/group. All values are presented as the means ± SEM. ANOVA, analysis of variance; ND, normal diet; WD, western diet; WT, wild-type.

Figure S5

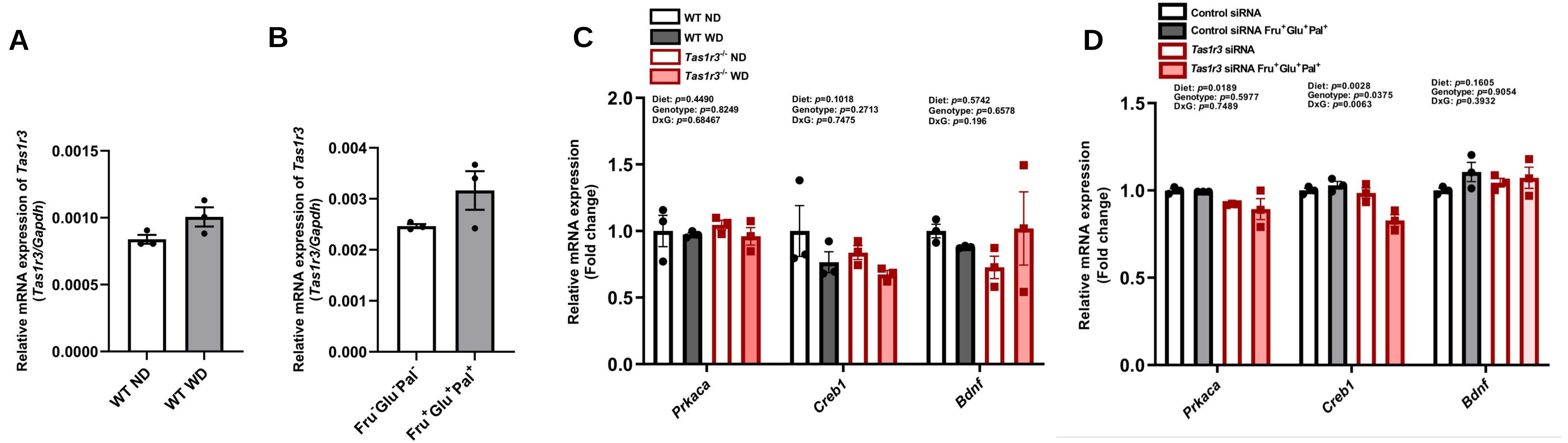

Additional file 1: Fig. S5. *Tas1r3*, *Prkaca*, *Creb1* and *Bdnf* mRNA expression in the hippocampal tissue and cultured adult hippocampal neuronal cell line.

(A) Relative mRNA expression of *Tas1r3* in hippocampal tissue of WT mice fed ND or WD for 12 weeks and (B) hippocampal neuronal cell line stimulated with or without fructose (10 mM), glucose (10 mM), and palmitate (10 μM). Unpaired two-tailed Student *t*-test. *n* = 3/group. (C) Relative mRNA expression of *Prkaca*, *Creb1* and *Bdnf* in hippocampal tissue of WT and *Tas1r3*$^{-/-}$ mice fed ND or WD for 12 weeks and (D) hippocampal neuronal cell line transfected with *Tas1r3* siRNA or scrambled control siRNA and stimulated with or without fructose (10 mM), glucose (10 mM), and palmitate (10 μM). Two-way ANOVA followed by Tukey's multiple comparison test. *n* = 3/group. All values are presented as the means ± SEM. ANOVA, analysis of variance; ND, normal diet; WD, western diet; WT, wild-type.
