# Supplementary material for: Taste receptor type 1 member 3 enables western diet-induced anxiety in mice
Source: BMC Biol. 2023 Nov 6;21:243. doi: 10.1186/s12915-023-01723-x (PMC10626698; doi:10.1186/s12915-023-01723-x)
Supplement: Supplementary file 2 — Additional file 2: Table S1. Cohorts of animals used for behavioral testing. Table S2. siRNA target sequences. Table S3. qPCR primers. [file 12915_2023_1723_MOESM2_ESM.docx]

**Table S1. Cohorts of animals used for behavioral testing.**

| **Sex** | **Cohort** | **Diet** | **Genotype** | **Numbers of animals** | **Test** |
| --- | --- | --- | --- | --- | --- |
| Male | **1** | ND | WT | 18 | OF, EPM |
|  |  |  | *Tas1r3^-/-^* | 14 |  |
|  |  | WD | WT | 24 |  |
|  |  |  | *Tas1r3^-/-^* | 21 |  |
|  | **2** | ND | WT | 9 | LDB, NSF |
|  |  |  | *Tas1r3^-/-^* | 9 |  |
|  |  | WD | WT | 11 |  |
|  |  |  | *Tas1r3^-/-^* | 11 |  |
| **Female** | **1** | ND | WT | 11 | OF, EPM |
|  |  |  | *Tas1r3^-/-^* | 14 |  |
|  |  | WD | WT | 19 |  |
|  |  |  | *Tas1r3^-/-^* | 15 |  |

Three independent cohorts were generated and used for the behavioral test. For each cohort, the table summarizes the number of animals per group and allocation to the experiments of interest. EPM, elevated plus maze; LDB, light-dark box; ND, normal diet; NSF, novelty suppressed feeding; OF, open field; WD, western diet; WT, wild-type.

**Table S2.** **siRNA target sequences.**

| **Target gene** | **Sense** | **Antisense** |
| --- | --- | --- |
| **Mouse**  ***Tas1r3*** | GACAUGGAAUAUGACCUGA | UCAGGUCAUAUUCCAUGUC |

**Table S3. qPCR primers.**

| **Gene** | **Forward primer sequence**  **(5′–3′)** | **Reverse primer sequence**  **(5′–3′)** |
| --- | --- | --- |
| **Mouse *Gapdh*** | AGGTCGGTGTGAACGGATTTG | TGTAGACCATGTAGTTGAGGTCA |
| **Mouse *Tas1r3*** | TGCTGCCTACTGCAACTACAC | CCGGTCACTTAGCCGATCC |
| **Mouse *Prkaca*** | CTAAAGCAGATCGAGCACACTC | GCCACCAGCTACATACTCCA |
| **Mouse**  ***Creb1*** | CCCAAAAACGAAGGGAAATCCT | CCTGGTGCATCAGAAGATAAGTC |
| **Mouse**  ***Bdnf*** | AAAGTCCCGGTATCCAAAGGCCAA | TAGTTCGGCATTGCGAGTTCCAGT |
| **Mouse *Dcx*** | AAACTGGAAACCGGAGTTGTC | CGTCTTGGTCGTTACCTGAGT |
| **Mouse *Map2*** | ACATCAAACATTCTGCTGGGGGCG | CAAGCGCCGCAGTGACATCCT |
| **Mouse *Sox11*** | CGACGACCTCATGTTCGACC | GACAGGGATAGGTTCCCCG |
| **Mouse *Agrp*** | CTCCGCGTCGCTGTGTAAG | GAAGCGGCAGTAGCACGTAG |
| **Mouse *Gabra2*** | GGACCCAGTCAGGTTGGTG | TCCTGGTCTAAGCCGATTATCAT |
| **Mouse *Bax*** | GGCGAATTGGAGATGAACTG | CAAAGTAGAAGAGGGCAACCAC |
| **Mouse *Bcl-2*** | TCGCCCTGTGGATGACTGA | CACTTGTGGCCCAGGTATG |
