# Supplementary figures and images for: Taste receptor type 1 member 3 enables western diet-induced anxiety in mice
Source: BMC Biol. 2023 Nov 6;21:243. doi: 10.1186/s12915-023-01723-x (PMC10626698; doi:10.1186/s12915-023-01723-x)

**Additional file 4: Fig. S6. Uncropped western blot figures.**

- Uncropped western blot Fig. 3F

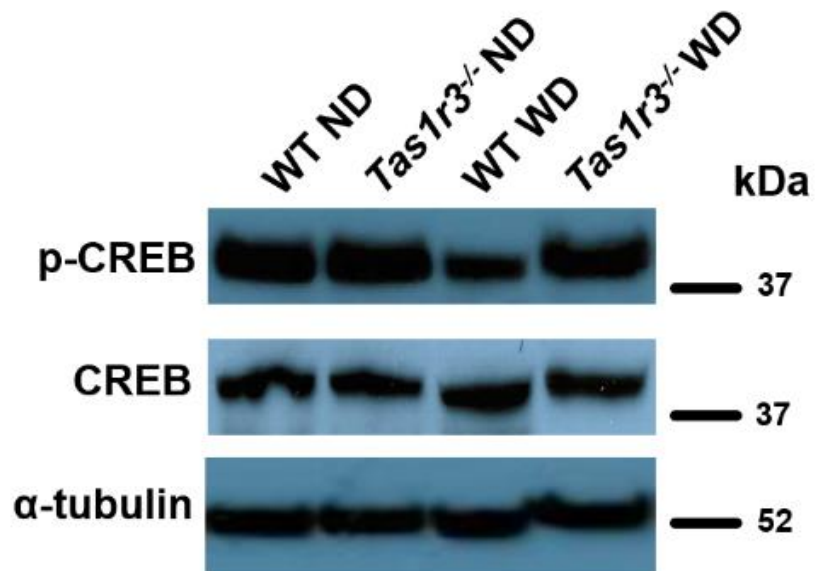

Supplement: Supplementary file 4 — Additional file 4: Fig. S6. Uncropped western blot figures. [file 12915_2023_1723_MOESM4_ESM.pdf]
